# Hem-o-Lok Clip in the First Part of Duodenum after Laparoscopic Cholecystectomy

**DOI:** 10.1155/2013/251634

**Published:** 2013-05-07

**Authors:** Mohammadreza Seyyedmajidi, Seyed Ashkan Hosseini, Shahin Hajiebrahimi, Jamshid Vafaeimanesh

**Affiliations:** ^1^Golestan Research Center of Gastroenterology and Hepatology-GRCGH, Golestan University of Medical Sciences, Gorgan, Iran; ^2^Clinical Research Development Center, Qom University of Medical Sciences, Qom 3719764799, Iran

## Abstract

Laparoscopic cholecystectomy (LC) and common bile duct exploration (LCBDE) have become the standard surgical procedure for cholecystolithiasis and choledocholithiasis. During the operation, cystic duct and vessels are usually controlled by Hem-o-Lok clips. We report a case with a complaint of severe abdominal pain for the previous 20 days. Her medical history was unremarkable except for laparoscopic cholecystectomy 8 months ago. In upper gastrointestinal endoscopy, two Hem-o-Lok clips at anterior wall of the first part of duodenum were detected. Therefore, the clip can migrate during postoperative period and Hem-o-Lok is not a very safe ligation method during laparoscopic cholecystectomy.

## 1. Introduction

There are several methods for the ligation of structures during minimally invasive operations. Many studies have examined the efficacy and safety of various devices in different situations. Each technique has potential drawbacks. Application of end loops requires dexterity and training. Titanium clips can slip from their primary position [1, 2]. New technology has played an important role in the proliferation of laparoscopy. A central issue that remains is meticulous hemostasis, particularly for larger vessels [2]. Despite their increasing popularity and widespread use, there has been a paucity of data when objectively comparing the relative safety and efficacy of these devices. There are potential disadvantages of energy-based instruments for hemostasis including lateral thermal spread, variable burst pressures, and the generation of smoke, vapor, and particulates which may compromise visibility [3].

**Figure 1:**
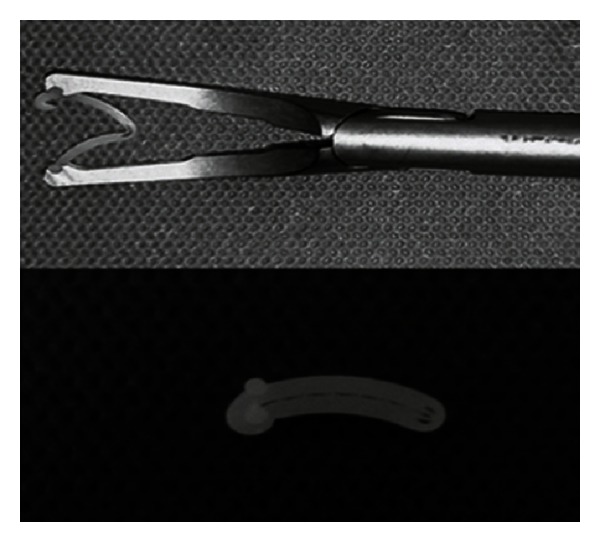
The Hem-o-Lok clip and its applier.

The Hem-o-Lok clip (Weck Closure Systems, Research Triangle Park, NC, USA) was introduced in 1999 (Figure 1). This nonabsorbable polymer clip has a lock engagement feature as well as teeth in the jaws that provide good security. In addition, recent experimental studies have tested the ability of the Hem-o-Lok to withstand pressures in comparison with other devices [2, 3]. This clip has gained popularity among laparoscopic urologists for the ligation of the renal hilum vessels during minimally invasive nephrectomy. Many others have also adopted the Hem-o-Lok for a variety of laparoscopic procedures in recent years [4].

Laparoscopic cholecystectomy (LC) and common bile duct exploration (LCBDE) have become the standard surgical procedure for cholecystolithiasis and choledocholithiasis. During the operation, cystic duct and vessels are usually controlled by Hem-o-Lok clips [5, 6]. Skeletonization of Calot's triangle and the controlling of gall bladder vessels and cystic duct successfully are the key points of the LC. Currently, Hem-o-Lok clips are generally used to ligate gall bladder vessels and cystic duct during LC. Besides, it is reported that Hem-o-Lok clips are safe to be used in other operations [7, 8]. 

In recent years, in few patients who underwent LC and LCBDE, clips were found in common bile duct (CBD) during fiberoptic choledochoscope, which is a dangerous finding. A very rare complication of laparoscopic cholecystectomy is clip migration with the formation of biliary stones around the clip. In a review of the published world literature within 1979–2008, sixty-nine well-documented cases of such complication were reported [1].

## 2. Case 

**Figure 2:**
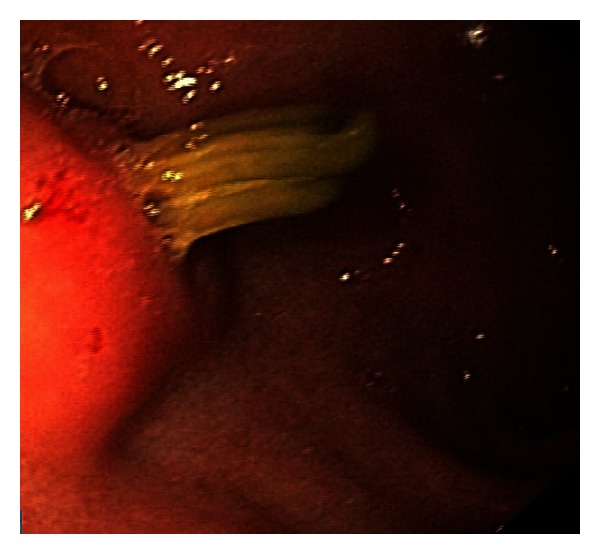
The Hem-o-Lok clips at anterior wall of the first part of duodenum.

**Figure 3:**
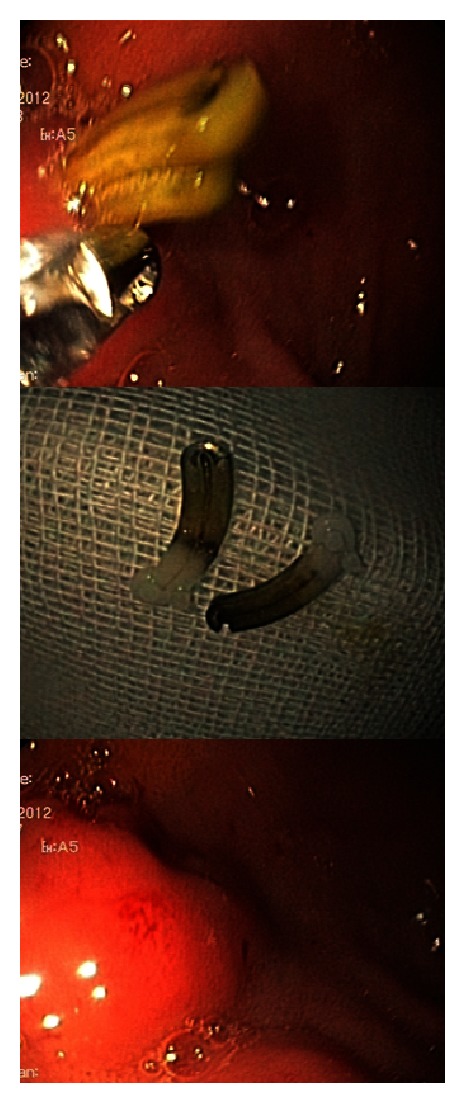
The Hem-o-Lok clips were removed using an Olympus grasping forceps.

A 41-year-old woman came with complaint of severe abdominal pain for the previous 20 days. Her medical history was unremarkable except for laparoscopic cholecystectomy 8 months before for symptomatic gall stone disease. On physical examination, there was tenderness in the epigastric area of abdomen. Her vital signs were normal. The complete blood count and clotting factors were normal. Blood tests were unremarkable for alanine aminotransferase, aspartate aminotransferase, alkaline phosphatase, total and direct bilirubin, serum amylase, and lipase level. The patient underwent upper gastrointestinal endoscopic (Olympus videoscope GIF-Q180, Japan) which demonstrated two Hem-o-Lok clips at anterior wall of the first part of duodenum (Figure 2). The Hem-o-Lok clips were removed using an Olympus grasping forceps (Figure 3). Eventually, the patient was discharged the same day without complications (hemorrhage or bile leakage).

## 3. Discussion 

The Hem-o-Lok clips have been a useful alternative to titanium clips, end loops, and endoscopic staplers [2]. This nonabsorbable polymer locking clip is inert, nonconductive, and compatible with CT scan and MRI. The lock engagement feature and the presence of teeth in the jaws provide good security. Loading of the applier with the clip is easy, and a flexible mechanism virtually prevents clips from falling out of the applier. There are several reports about the safety of Hem-o-Lok clips in minimally invasive operations [1–4].

The MAUDE database is a reporting system mandated by the FDA for the surveillance of medical devices. It includes reports of adverse events involving medical devices that occur after device approval. MAUDE has been transformed into a searchable online database [9]. However, these reports to the FDA are voluntary, and it is possible that a significant number of events are never reported. Additionally, attempts to quantify the occurrence of adverse events as a risk percentage are limited, because the absolute number of procedures performed by any given device is not tracked by the FDA and is unavailable in the MAUDE database [10]. Accordingly, the FDA website states that “MAUDE data are not intended to be used either to evaluate rates of adverse events or to compare adverse event occurrence rates across devices” [11].

Migration of the clip into the CBD and the resulting stone formation is a rare but well-recognized complication of cholecystectomy. Recently, V. H. Chong and C. F. Chong published a review of 69 publications reporting 80 such cases. Metal clips were used in all cases except for 2, for which absorbable clips were used. The median time from cholecystectomy to clinical presentation of the migrated clip was 26 months (range: 11 days to 20 years). Most of the patients presented with typical symptoms of CBD stone, and endoscopic removal was successful in most cases [8]. Yahui et al. found clips dropping into CBD in 8 patients during fiberoptic choledochoscope 2-3 months after operation. Therefore, there is a risk that a clip can migrate during postoperative period, which will probably lead to hemorrhage and bile leakage after LC if this occurs within postoperative few days or will even cause recurrent stone in CBD [5]. As a matter of fact, the experience shows that a clip can drop. The reason may be a rejection response of human body [5]. Also, there are several reports of the Hem-o-Lok clip migration during radical prostatectomy into the rectum and urinary bladder, with subsequent bladder stone formation [12–16].

To our knowledge, Hem-o-Lok clip migration after cholecystectomy into the first part of duodenum has not been previously reported. The anatomic basis of cystic duct ligation site close to duodenum can possibly lead to clip fistula formation into duodenum by rejection-response mechanism. Properly applied Hem-o-Lok clips during basic laparoscopic procedures are a safe option for the ligation of the structures. Surgeons must be educated regarding its proper application. The complications of this method are rare and Hem-o-Lok is a safe ligation method during LC to control cystic duct and vessels. 

## 4. Conclusion

To our knowledge, Hem-o-Lok clip migration after cholecystectomy into the first part of duodenum has not been previously reported. The anatomic basis of cystic duct ligation site close to duodenum can possibly lead to clip fistula formation into duodenum by rejection-response mechanism. Thus, Hem-o-Lok is not a very safe ligation method during laparoscopic cholecystectomy.
